# Implications of coronary calcification on the assessment of plaque pathology: a comparison of computed tomography and multimodality intravascular imaging

**DOI:** 10.1007/s00330-024-10996-x

**Published:** 2024-08-22

**Authors:** Nathan Angelo Lecaros Yap, Anantharaman Ramasamy, Ibrahim Halil Tanboga, Xingwei He, Murat Cap, Retesh Bajaj, Medeni Karaduman, Ajay Jain, Pieter Kitslaar, Alexander Broersen, Xiaotong Zhang, Hessam Sokooti, Johan H. C. Reiber, Jouke Dijkstra, Mick Ozkor, Patrick W. Serruys, James C. Moon, Anthony Mathur, Andreas Baumbach, Ryo Torii, Francesca Pugliese, Christos V. Bourantas

**Affiliations:** 1 Department of Cardiology, Barts Heart Centre, Barts Health NHS Trust, London, UK; 2 Centre for Cardiovascular Medicine and Devices, William Harvey Research Institute, Queen Mary University London, London, UK; 3 Department of Biostatistics and Cardiology, Nisantasi University Medical School, Istanbul, Turkey; 4 Department of Cardiology, Yuzuncu Yil University, Van, Turkey; 5 Division of Image Processing, Department of Radiology, Leiden University Medical Center, Leiden, The Netherlands; 6 Medis Medical Imaging Systems, Leiden, The Netherlands; 7 Faculty of Medicine, National Heart & Lung Institute, Imperial College London, London, UK; 8 Department of Cardiology, National University of Ireland, Galway, Ireland; 9 Institute of Cardiovascular Sciences, University College London, London, UK; 10 Department of Mechanical Engineering, University College London, London, UK

**Keywords:** Computed tomography angiography, Vascular calcification, Atherosclerosis, Near-infrared spectroscopy intravascular ultrasound

## Abstract

**Objectives:**

This study aimed to investigate the impact of calcific (Ca) on the efficacy of coronary computed coronary angiography (CTA) in evaluating plaque burden (PB) and composition with near-infrared spectroscopy-intravascular ultrasound (NIRS-IVUS) serving as the reference standard.

**Materials and methods:**

Sixty-four patients (186 vessels) were recruited and underwent CTA and 3-vessel NIRS-IVUS imaging (NCT03556644). Expert analysts matched and annotated NIRS-IVUS and CTA frames, identifying lumen and vessel wall borders. Tissue distribution was estimated using NIRS chemograms and the arc of Ca on IVUS, while in CTA Hounsfield unit cut-offs were utilized to establish plaque composition. Plaque distribution plots were compared at segment-, lesion-, and cross-sectional-levels.

**Results:**

Segment- and lesion-level analysis showed no effect of Ca on the correlation of NIRS-IVUS and CTA estimations. However, at the cross-sectional level, Ca influenced the agreement between NIRS-IVUS and CTA for the lipid and Ca components (*p*-heterogeneity < 0.001). Proportional odds model analysis revealed that Ca had an impact on the per cent atheroma volume quantification on CTA compared to NIRS-IVUS at the segment level (*p*-interaction < 0.001). At lesion level, Ca affected differences between the modalities for maximum PB, remodelling index, and Ca burden (*p*-interaction < 0.001, 0.029, and 0.002, respectively). Cross-sectional-level modelling demonstrated Ca’s effect on differences between modalities for all studied variables (*p*-interaction ≤ 0.002).

**Conclusion:**

Ca burden influences agreement between NIRS-IVUS and CTA at the cross-sectional level and causes discrepancies between the predictions for per cent atheroma volume at the segment level and maximum PB, remodelling index, and Ca burden at lesion-level analysis.

**Clinical relevance statement:**

Coronary calcification affects the quantification of lumen and plaque dimensions and the characterization of plaque composition coronary CTA. This should be considered in the analysis and interpretation of CTAs performed in patients with extensive Ca burden.

**Key Points:**

- *Coronary CT Angiography is limited in assessing coronary plaques by resolution and blooming artefacts*.
- *Agreement between dual-source CT angiography and* NIRS-IVUS *is affected by a Ca burden for the per cent atheroma volume*.
- *Advanced CT imaging systems that eliminate blooming artefacts enable more accurate quantification of coronary artery disease and characterisation of plaque morphology*.

## Introduction

Computed tomography coronary angiography (CTA) is regarded as the first-line diagnostic modality for assessing the extent and severity of coronary artery disease (CAD). Cumulative data has shown that CTA has a high negative predictive value in excluding obstructive CAD, but it has limited efficacy in assessing plaque pathophysiology and vulnerability, especially in patients with CAD [1–4]. This has been attributed to the limited resolution of CTA compared to intravascular imaging, as well as to the blooming artefacts that are seen in calcified lesions that often preclude the accurate delineation of the lumen and vessel wall borders on CTA.

Several studies have attempted to examine the implications of coronary calcification on the performance of CTA in quantifying the extent and severity of CAD using intravascular imaging as a reference standard [1–3]. However, most of these reports relied on retrospective analysis of acquired data and focused only on the quantification of the lumen dimensions [1] or the performance of the modality in assessing lesion severity [4, 5] and calcific (Ca) burden [2], and did not provide a holistic evaluation of the implications of coronary calcification in the performance of CTA in measuring plaque burden (PB) and characterising plaque composition.

## Methods

### Study population

All the patients included in the study signed a consent form prior to recruitment; the study was approved by the local ethics committee (REC reference: 17/SC/0566) and was conducted in accordance with the Declaration of Helsinki. The present study is a post-hoc analysis of a recently completed prospective study that aimed to evaluate the performance of CTA in assessing coronary artery morphology and physiology using multimodality near-infrared spectroscopy-intravascular imaging (NIRS-IVUS) as reference standard (Clinicaltrials.gov NCT03556644). The study design and objectives have been described previously [6]. In brief, 70 patients with a chronic coronary syndrome that had a coronary angiogram showing significant CAD, requiring treatment with percutaneous coronary intervention (PCI) or further evaluation with invasive imaging of functional assessment were recruited and underwent CTA before being listed for repeat invasive angiogram + /− PCI and 3 vessel NIRS-IVUS imaging. The CTA and NIRS-IVUS data acquisition protocol is described in the supplementary file. Patients were recruited for the study between March 2018–July 2019

### CTA and NIRS-IVUS data analysis

An interventional cardiologist with expertise in CTA analysis reviewed the NIRS-IVUS and CTA data and identified the most proximal and distal side branch in NIRS-IVUS that was visible in CTA and used this to define a segment of interest, i.e. a segment that was assessed by both modalities. Segments with poor image quality, significant artefacts, and those portraying a stented vessel were excluded from the analysis.

CTA analysis was performed in the segment of interest by an expert analyst with an established reproducibility blinded to NIRS-IVUS analysis using commercially available software (QAngioCT Research Edition 3.1, Medis Medical Imaging Systems) [7]. In each CTA cross-section within the segment of interest, the analyst manually drew the lumen and vessel wall borders. The established Hounsfield unit cut-off proposed by de Graaf et al, [8] incorporated in the software was used to characterise plaque composition in each CTA cross section and to define four tissue types, fibrotic tissue (FT), fibrofatty (FF), necrotic core (NC) and Ca tissue. These cutoffs are based on a histology study that included CTA data acquired with a tube voltage of 120 kV but phantom studies indicate that they also apply to CTA images obtained at 100 kV [9, 10]. The detected tissue types on CTA were used to generate spread-out plots of NC and Ca distribution with the *X*-axis indicating the longitudinal and the *Y*-axis the circumferential position of these plaque components, which were portrayed in yellow and semi-transparent white color, while the FT and the FF were shown in red color [11]. In these spread-out plots, the Ca and lipid core burden index (CaBI, LCBI) were estimated and defined as the number of semi-transparent white or yellow pixels indicating the presence of Ca and NC divided by the total number of pixels multiplied by 1000. Moreover, the maxLCBI_4mm_ was defined as the maximum LCBI value in a 4 mm segment.

**Fig. 1:**
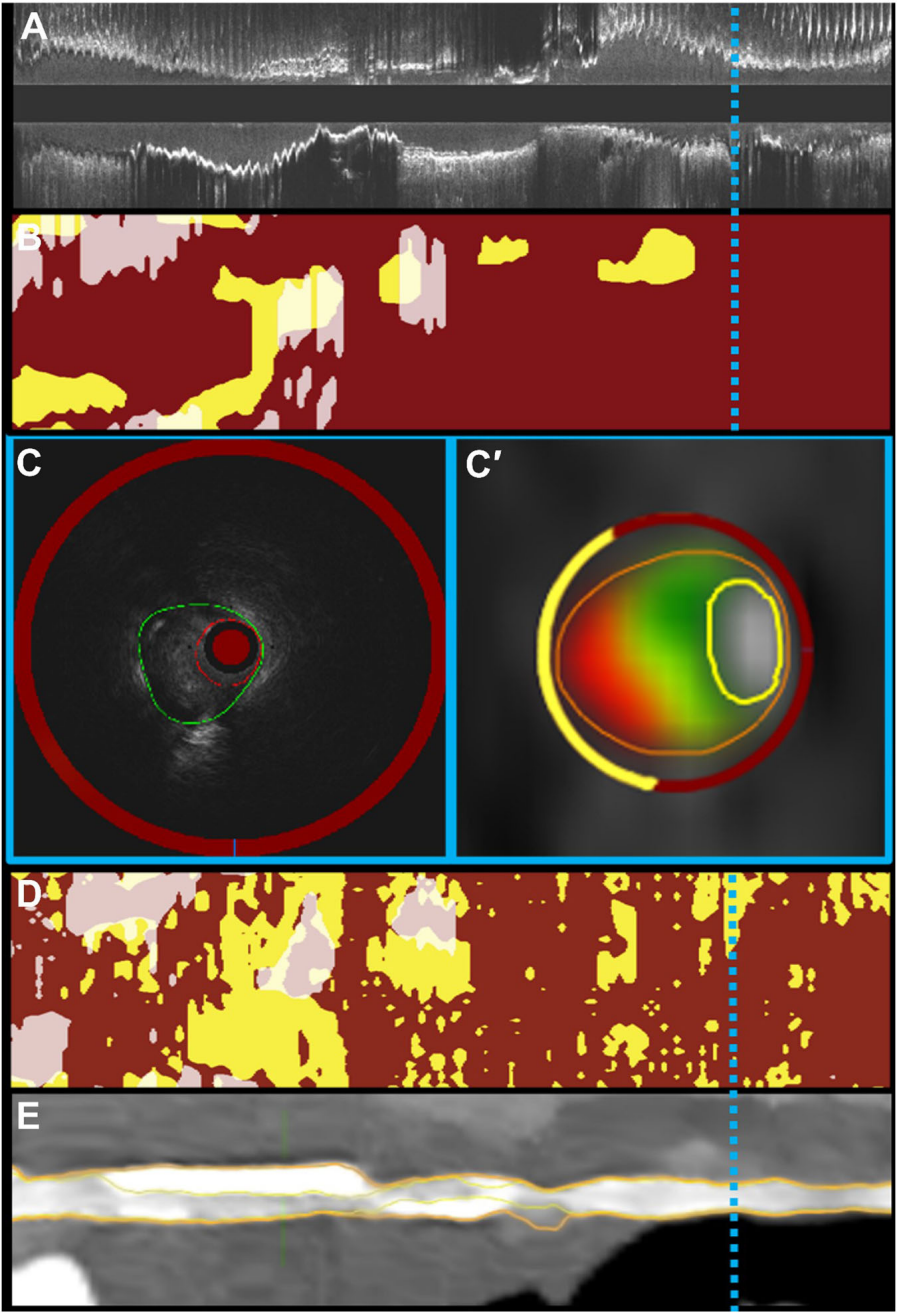
Typical example of tissue distribution in NIRS-IVUS and CTA imaging data. **A** Displays a longitudinal section of the IVUS sequence that was matched with the CTA images obtained from a left anterior descending coronary artery. **B** Shows a spread-out plot of the circumferential and longitudinal distribution of the lipid (indicated with yellow color) and Ca (displayed in a semi-transparent white color) tissue in the studied vessel while **C**, **C**” corresponding NIRS-IVUS and CTA cross sections with the estimations of the two modalities about plaque composition. **D** Illustrates tissue components distribution in a spread-out plot on the CTA imaging data and **E** is a longitudinal section of the studied vessel with the annotated lumen and vessel borders in CTA

NIRS-IVUS segmentation was performed for the segment of interest by another expert analyst with established reproducibility blinded to CTA analysis [12]. NIRS-IVUS analysis included the extraction of the end-diastolic frames using a validated deep learning methodology and, in these frames, the lumen and external elastic membrane (EEM) borders were manually annotated [13]. In addition, in these frames, the circumferential distribution of the Ca was manually annotated with an arc and these annotations, together with the lipid distribution derived from the NIRS chemogram, were used to generate spread-out plots of Ca and lipid core distribution with the *X*-axis indicating the longitudinal and the *Y*-axis the circumferential location of these components. Similarly, in the spread-out plots generated from the CTA data, FT was marked as red, semi-transparent white for the Ca and yellow color for the lipid core tissue; these plots were used to calculate the LCBI, maxLCBI_4mm_, and the CaBI (Fig. 1).

The CTA and NIRS-IVUS images were co-registered using a methodology described in the supplementary file and the estimations of the two modalities were compared.

### Implications of coronary calcification on the performance of CTA in assessing plaque pathology

The effect of coronary calcification on the performance of CTA in assessing plaque pathology was examined at segment-, lesion- and cross-sectional-level.

- Segment-level analysis: the mean Ca area was estimated for each segment of interest defined as: Ca volume/segment length and this was used to split the studied segments in terciles. In each tercile, we compared the estimations of NIRS-IVUS and CTA for the lumen, vessel, total atheroma volume (TAV, defined as vessel—lumen volume) and percentage atheroma volume (PAV, defined as: 100 × TAV/vessel volume), as well as for the CaBI, LCBI, and maxLCBI_4mm_. Moreover, we examined the effect of mean Ca area, and arc (defined as 360 × CaBI/1000) on the association between NIRS-IVUS and CTA measurements for the entire segment dataset.
- Lesion-level analysis: lesions in NIRS-IVUS as three consecutive end-diastolic frames with a PB ≥ 40%. Two lesions were considered separate if there was a segment between these two lesions with no significant atheroma (PB < 40%) with a length ≥ 5 mm [14, 15]. The correspondence between NIRS-IVUS and CTA cross-sections—defined using dedicated software—was used to identify the location of the lesions detected by NIRS-IVUS on CTA and these segments were classified as lesions in CTA. In each lesion, the mean Ca area was computed, and this was used to split the lesions in terciles. In each tercile, we compared the estimations of CTA and NIRS-IVUS for the minimum lumen area (MLA), maximum PB, reference lumen and vessel area and the remodelling index, as well as the CaBI, LCBI, maxLCBI_4mm_. Moreover, we examined the effect of mean Ca area and arc on the agreement between CTA and NIRS-IVUS for the entire lesion dataset.
- Cross-sectional-level analysis: the Ca area of each CTA cross-section was used to split the matched NIRS-IVUS and CTA cross-section terciles. In each tercile, we compared the estimations of the two modalities for the lumen, vessel, plaque area, PB, CaBI, and LCBI (indicated by the Ca and lipid arc). In addition, we assessed the effect of the Ca area and arc on the association between NIRS-IVUS and CTA estimations for the above metrics.

### Statistical analysis

Continuous variables are presented as median (interquartile range), while categorical as absolute values and percentages. The estimations of NIRS-IVUS and CTA were compared using Wilcoxon signed-rank tests. Bland–Altman (BA) analysis was employed to assess bias and the limits of agreements (LOA) between CTA and NIRS-IVUS measurements. Non-parametric BA analysis was chosen for the segment- and lesion-level analysis due to the small sample size and the non-normal distribution of differences, while parametric analysis was used for cross-sectional level analysis due to the larger sample size. In the parametric BA analysis, bias was calculated as the mean difference between the two methods, while the LOA was defined as a range centred around the mean bias ± 1.96 × standard deviation. For the non-parametric BA analysis, the median bias and the 2.5th–97.5th percentiles were computed using quantile regression.

The agreement between the two modalities was tested using intra-class correlation coefficients (ICC) under a two-way random effects model, considering absolute agreement and average measurements using a linear mixed model. First, the unadjusted ICC was estimated: subjects and modalities (NIRS-IVUS/CT) were included as random effects in the model. Subsequently, to obtain the adjusted ICC, the mean Ca area/mean Ca arc were included as fixed covariates in the model (using three knots-restricted cubic spline transformations for lesion- and segment-analysis and using 5 knots-restricted cubic spline transformations for frame-level data). When the confidence intervals of the unadjusted and adjusted ICCs overlapped, the comparative *p*-value was considered non-significant. For sensitivity analysis, the mean Ca area was divided into terciles and deciles and then ICC was calculated for each group. To detect the presence of heterogeneity among the terciles for ICCs, a meta-analysis was conducted using a tau-based heterogeneity *p*-value.

Finally, proportional odds models (for both segment- and lesion-level data) and ordinary least square models (for cross-sectional-level data) were used to estimate the differences between the predictions of NIRS-IVUS and CTA for lumen and vessel wall dimensions and PB and composition. The models were constructed both with (adjusted differences) and without the inclusion of the Ca area (unadjusted differences). The interaction between the Ca area on CTA and NIRS-IVUS measurements was assessed to examine whether the estimated differences between CTA and NIRS-IVUS measurements are influenced by the Ca area on CTA and explore whether the performance of the CTA to predict the NIRS-IVUS measurements varies with the changes in Ca.

All statistical tests were two-tailed, and statistical significance was set at *p* < 0.05; analyses were performed in the R software v. 4.2.2 (R statistical software) using “lme4”, “ppcor”, “dplyr”, “rms”, “meta” and “ggplot2” packages.

## Results

### Studied patients

Of the 70 patients recruited in the study, 65 patients had NIRS-IVUS and CTA imaging. In one case, a malignancy was found on CTA and the patient did not have a cardiac catheterization, one patient developed acute kidney injury post CTA and was excluded from the study, and in three cases there was NIRS-IVUS catheter malfunction and intravascular imaging was not performed. Moreover, 1 patient was excluded because of poor CTA image quality caused by an increased Ca burden that did not allow CTA segmentation. From the remaining patients, 9 additional vessels were excluded because of motion artefacts that prohibited frame-by-frame NIRS-IVUS and CTA matching. Therefore, in total, 64 patients, and 186 vessels with interpretable CTA and NIRS-IVUS imaging data (23,605 matched cross-sections) were included in the final analysis. The baseline characteristics of the patients included in the study are shown in Supplementary Table S1.

### Segment-level analysis

Segment-level analysis results are shown in Table 1. CTA underestimated the lumen and vessel volume, TAV, PAV, and CaBI compared to NIRS-IVUS, but there was no difference between the two modalities for LCBI and maxLCBI_4mm_ although the LOA was large. Results were consistent in the 3 terciles with the only difference in the CaBI in the high Ca area tercile where there was no difference between the two groups (Table 1).

**Table 1:** Segment-level comparison of the estimations of NIRS-IVUS and CTA in the entire dataset and in the low, intermediate and high Ca area groups

	NIRS-IVUS	CTA	*p-*value *	Median bias (95% CI)
Entire set of studied segments				
Lumen volume, mm^3^	420.39 (223.82, 612.78)	309.20 (176.73, 463.81)	< 0.001	89.43 (1.26, 251.68)
Vessel volume, mm^3^	735.01 (385.20, 1059.41)	454.59 (247.49, 672.14)	< 0.001	251.32 (42.81, 573.23)
TAV, mm^3^	292.80 (149.37, 465.17)	116.09 (64.87, 229.72)	< 0.001	139.93 (9.57, 368.73)
Percent atheroma volume, %	41.06 (33.75, 47.85)	27.67 (20.61, 37.17)	< 0.001	10.60 (− 1.34, 23.91)
LCBI	33.87 (4.77, 91.15)	28.36 (4.11, 78.62)	0.758	0 (− 121.09, 96.91)
CaBI	59.35 (16.51, 131.40)	50.81 (9.95, 111.66)	< 0.001	11.27 (− 43.62, 78.94)
MaxLCBI_4mm_	228.12 (60.00, 407.19)	230.00 (33.44, 538.75)	0.242	− 10 (− 537.50, 343.75)
Low Ca area tercile				
Lumen volume, mm^3^	287.39 (153.62, 502.66)	216.02 (132.14, 375.12)	< 0.001	87.94 (− 0.12, 239.94)
Vessel volume, mm^3^	468.81 (246.43, 845.13)	319.13 (166.07, 521.45)	< 0.001	147.16 (20.36, 573.23)
TAV, mm^3^	151.91 (92.51, 343.44)	63.33 (37.08, 129.92)	< 0.001	80.59 (22.17, 361.04)
Percent atheroma volume, %	37.99 (30.82, 42.70)	21.65 (18.54, 25.64)	< 0.001	13.77 (2.34, 26.00)
LCBI	16.13 (2.01, 52.58)	19.39 (1.01, 64.83)	0.085	− 4.77 (− 137.79, 62.24)
CaBI	13.53 (1.02, 42.46)	3.19 (0.00, 12.50)	< 0.001	5.99 (− 3.99, 53.18)
MaxLCBI_4mm_	173.75 (44.38, 310.94)	141.88 (11.01, 530.00)	0.078	− 41.25 (− 530.00, 248.75)
Intermediate Ca area tercile				
Lumen volume, mm^3^	461.85 (275.35, 664.55)	355.44 (219.38, 496.04)	< 0.001	105.72 (9.23, 261.63)
Vessel volume, mm^3^	792.43 (544.06, 1106.64)	523.01 (317.21, 661.53)	< 0.001	319.97 (76.856, 586.07)
TAV, mm^3^	315.25 (184.06, 486.80)	111.83 (76.01, 188.82)	< 0.001	174.06 (42.53, 386.64)
Percent atheroma volume, %	41.32 (33.50, 48.99)	26.04 (20.32, 31.04)	< 0.001	13.10 (3.23, 25.87)
LCBI	22.49 (1.98, 63.93)	9.96 (1.73, 51.43)	0.112	1.68 (− 65.23, 102.44)
CaBI	58.52 (34.11, 102.46)	53.07 (30.75, 76.07)	0.001	17.64 (− 27.45, 77.30)
MaxLCBI_4mm_	184.38 (45.00, 340.31)	140.62 (20.00, 385.00)	0.754	0 (− 427.50, 343.75)
High Ca area tercile				
Lumen volume, mm^3^	441.49 (267.54, 572.41)	336.96 (216.94, 466.19)	< 0.001	86.53 (14.45, 242.89)
Vessel volume, mm^3^	776.69 (537.17, 1073.69)	573.94 (378.55, 772.33)	< 0.001	252.891 (48.33, 509.59)
TAv, mm^3^	344.86 (241.61, 522.55)	227.51 (128.34, 321.65)	< 0.001	157.546 (− 10.19, 327.07)
Percent atheroma volume, %	45.52 (39.07, 49.18)	37.97 (32.82, 45.70)	< 0.001	6.75 (− 8.99, 17.76)
LCBI	55.77 (25.81, 114.65)	65.13 (21.02, 138.40)	0.669	3.18 (− 175.72, 110.66)
CaBI	146.72 (93.53, 245.42)	139.34 (105.04, 200.22)	0.220	19.30 (− 111.68, 139.77)
MaxLCBI_4mm_	332.50 (188.75, 466.88)	323.12 (158.75, 612.19)	0.488	− 2.5 (− 560.00, 375.00)

*CaBI* calcific burden index, *CTA* computed tomography coronary angiography, *LCBI* lipid core burden index, *NIRS-IVUS* near-infrared spectroscopy intravascular ultrasound, *MaxLCBI*_*4mm*_ maximum LCBI in 4 mm segment

* *p-*values were obtained by the Wilcoxon signed rank test

**Fig. 2:**
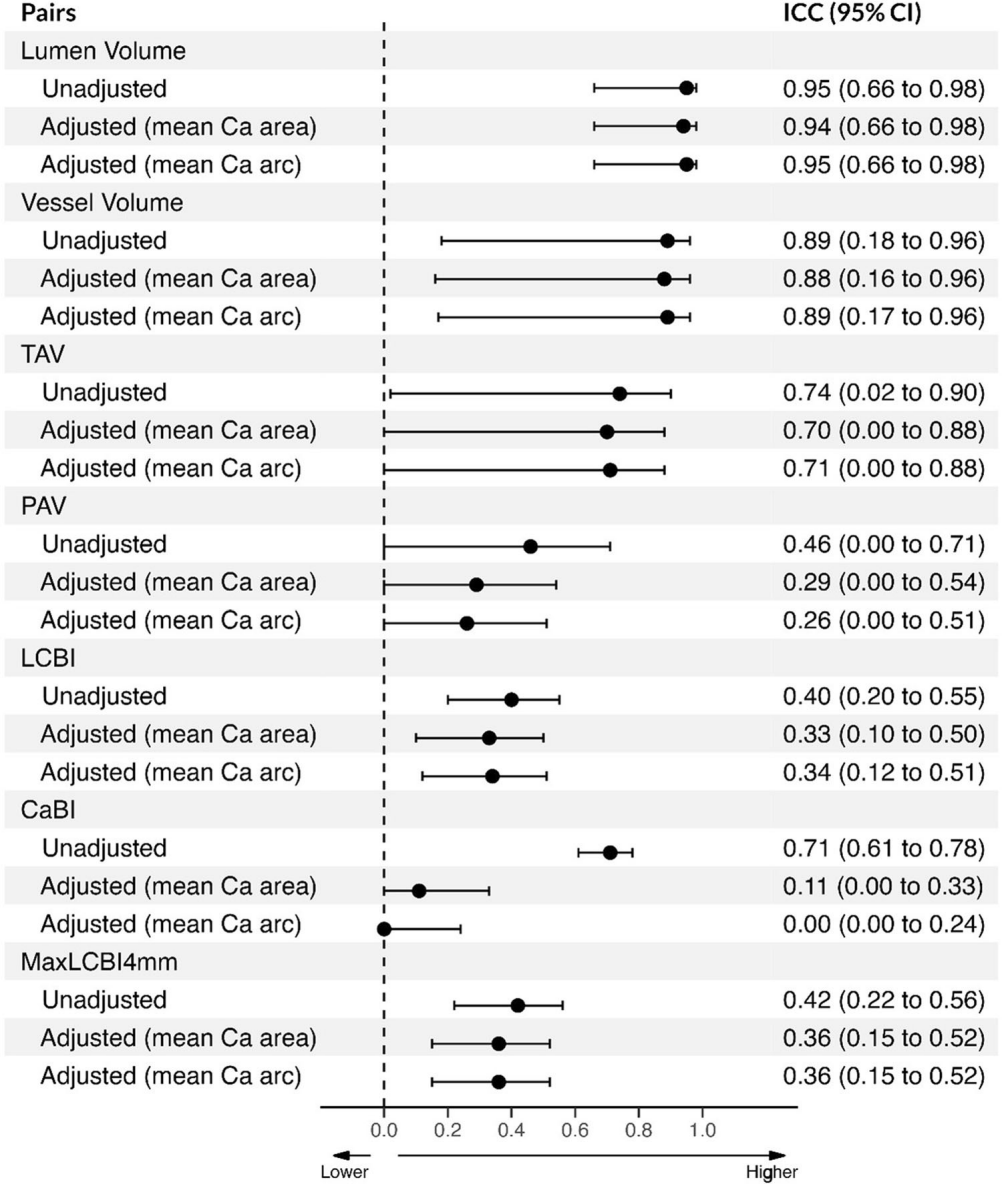
Unadjusted and adjusted (by mean Ca area and mean ca arc) ICC and 95% CI for measurements between NIRS-IVUS and CTA estimated by linear mixed model at segment level

**Fig. 3:**
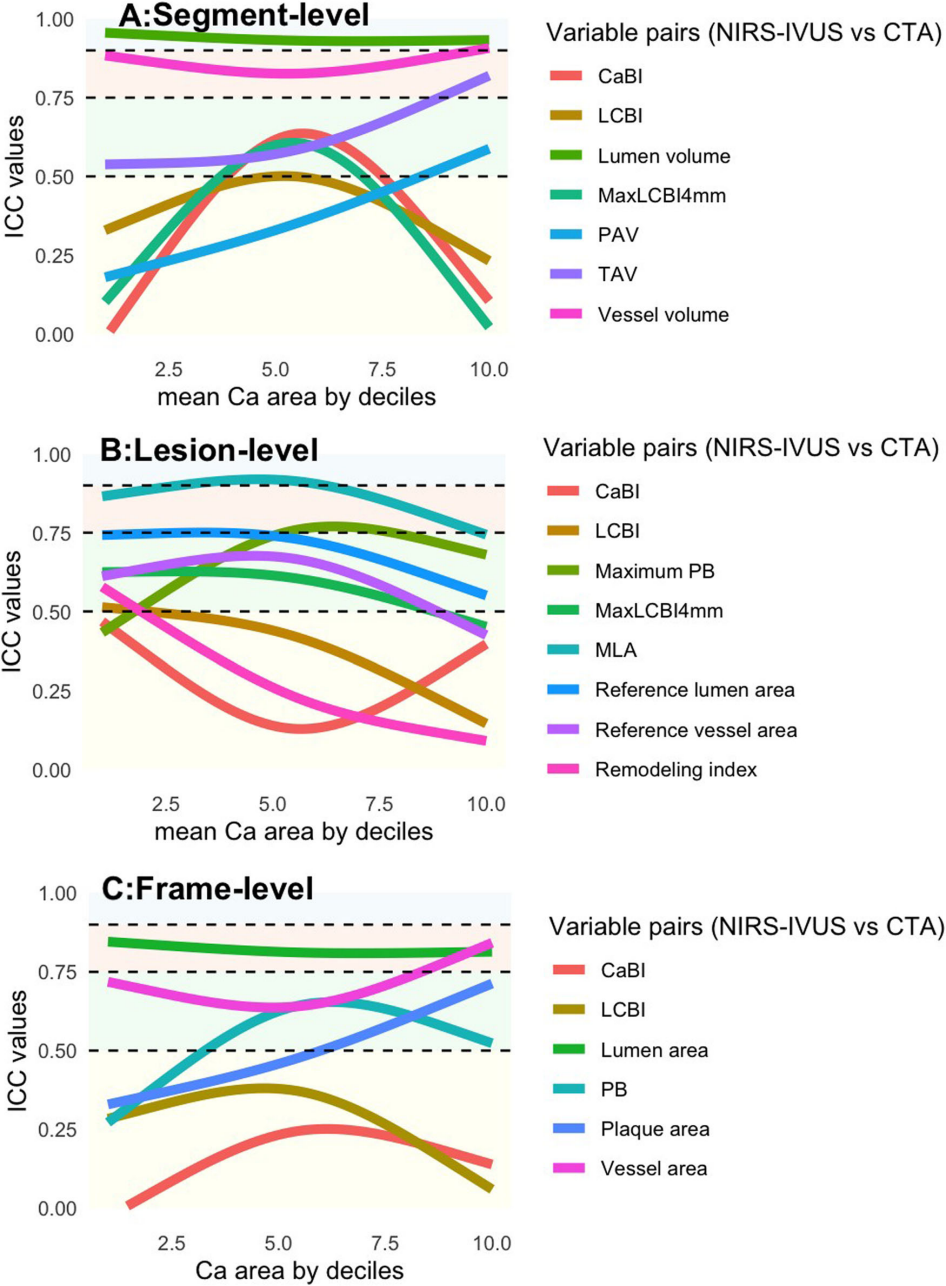
ICC for measurements between NIRS-IVUS and CTA according to Ca area deciles at segment (**A**), lesion- (**B**), and frame-level analysis (**C**). Horizontal black dashed lines have been added to facilitate the interpretation of ICC, and they represent 0.50, 0.75, and 0.90 (< 0.50 indicating poor, 0.50–0.75 indicating moderate, 0.75–0.90 indicating good, and > 0.90 indicating excellent agreement). Only ICC points according to Ca area tertiles are shown on the plot, 95% CI is not displayed

The unadjusted ICCs between NIRS-IVUS and CTA are shown in Fig. 2; the ICC was high for the lumen and vessel volume, moderate for the TAV and CaBI, and weak for the PAV, LCBI, and maxLCBI_4mm_. After adjusting for the mean Ca area or mean Ca arc, the ICC for the CaBI was significantly lower but there was no difference for other variables (Fig. 2). The effect of an increasing mean Ca area on the association of NIRS-IVUS and CTA at segment-level is illustrated in Fig. 3A. Overall, there was no statistically significant difference between the ICC values for the estimations of NIRS-IVUS and CTA in the 3 Ca area groups (Table 2 and Supplementary Fig. 1).

**Table 2:** ICC values between NIRS-IVUS and CTA estimations at segment-, lesion- and cross-sectional-level in the different Ca area terciles

	Low Ca tercile ICC	*p*-value	Intermediate Ca tercile ICC	*p*-value	High Ca tercile ICC	*p*-value	Heterogeneity *p*-value
Segment-level analysis							
Lumen volume	0.95 (0.72, 0.98)	< 0.001	0.94 (0.55, 0.98)	< 0.001	0.94 (0.65, 0.98)	< 0.001	0.993
Vessel volume	0.87 (0.27, 0.96)	< 0.001	0.88 (−0.05, 0.97)	< 0.001	0.90 (0.28, 0.97)	< 0.001	0.994
TAV	0.53 (− 0.08, 0.77)	< 0.001	0.70 (− 0.16, 0.89)	< 0.001	0.79 (0.13, 0.92)	< 0.001	0.669
PAV	0.19 (− 0.16, 0.48)	0.021	0.37 (− 0.21, 0.69)	< 0.001	0.50 (0.15, 0.70)	0.001	0.363
LCBI	0.27 (− 0.19, 0.55)	0.105	0.55 (0.26, 0.73)	0.001	0.25 (− 0.24, 0.55)	0.129	0.281
CaBI	0.36 (− 0.03, 0.61)	0.015	0.65 (0.39, 0.80)	< 0.001	0.25 (− 0.24, 0.55)	0.130	0.111
MaxLCBI_4mm_	0.36 (− 0.04, 0.61)	0.036	0.55 (0.26, 0.73)	0.001	0.18 (− 0.35, 0.51)	0.217	0.290
Lesion-level analysis							
MLA	0.93 (0.75, 0.97)	< 0.001	0.90 (0.65, 0.96)	< 0.001	0.79 (0.31, 0.91)	< 0.001	0.692
Maximum PB	0.45 (− 0.22, 0.75)	< 0.001	0.74 (0.46, 0.86)	< 0.001	0.74 (0.56, 0.83)	< 0.001	0.529
Reference lumen area	0.82 (0.04, 0.94)	< 0.001	0.78 (0.33, 0.90)	< 0.001	0.65 (− 0.09, 0.85)	< 0.001	0.858
Reference vessel area	0.73 (− 0.16, 0.91)	< 0.001	0.74 (− 0.15, 0.91)	< 0.001	0.52 (− 0.23, 0.81)	< 0.001	0.802
Remodelling index	0.47 (0.19, 0.65)	0.002	0.02 (− 0.53, 0.37)	0.470	0.14 (− 0.34, 0.45)	0.253	0.128
LCBI	0.59 (0.38, 0.73)	< 0.001	0.41 (0.10, 0.61)	0.008	0.24 (− 0.15, 0.50)	0.098	0.147
CaBI	0.63 (0.39, 0.77)	< 0.001	0.31 (− 0.04, 0.54)	0.037	0.60 (0.39, 0.74)	< 0.001	0.155
MaxLCBI_4mm_	0.61 (0.35, 0.76)	< 0.001	0.61 (0.39, 0.75)	< 0.001	0.54 (0.29, 0.70)	< 0.001	0.863
Cross-sectional-level analysis							
Lumen area	0.89 (0.63, 0.95)	< 0.001	0.82 (0.12, 0.93)	< 0.001	0.82 (0.35, 0.92)	< 0.001	0.883
Vessel area	0.75 (− 0.10, 0.91)	< 0.001	0.66 (− 0.21, 0.88)	< 0.001	0.76 (0.05, 0.90)	< 0.001	0.961
Plaque area	0.43 (− 0.09, 0.68)	< 0.001	0.42 (− 0.16, 0.69)	< 0.001	0.68 (0.42, 0.80)	< 0.001	0.345
PB	0.35 (− 0.07, 0.58)	< 0.001	0.47 (− 0.05, 0.70)	< 0.001	0.67 (0.65, 0.69)	< 0.001	0.999
LCBI	0.27 (0.24, 0.29)	< 0.001	0.36 (0.32, 0.39)	< 0.001	0.20 (0.15, 0.25)	< 0.001	< 0.001
CaBI	0.00 (− 0.03, 0.04)	0.416	0.20 (0.15, 0.24)	< 0.001	0.46 (0.44, 0.51)	< 0.001	< 0.001

*CaBI* calcific burden index, *CTA* computed tomography coronary angiography, *LCBI* lipid core burden index, *MaxLCBI*_*4mm*_ maximum LCBI in 4 mm segment, *MLA* minimum lumen area, *PAV* percentage atheroma volume, *PB* plaque burden, *TAV* total atheroma volume

Both unadjusted and adjusted by mean Ca area proportional odds model showed that the estimated median lumen volume, vessel volume, TAV and PAV were larger on NIRS-IVUS than the CTA. There was no significant interaction between the mean Ca area and lumen and vessel volume but there was a borderline interaction (*p* = 0.070) for the TAV and a significant interaction for the PAV (*p*-interaction < 0.001). No significant differences were noted between NIRS-IVUS and CTA for the estimated CaBI, LCBI, and maxLCBI_4mm_ measurements in both unadjusted and adjusted models. For these variables, there was no interaction between the two modalities and the mean Ca area (Supplementary Fig. 2).

### Lesion-level analysis

**Fig. 4:**
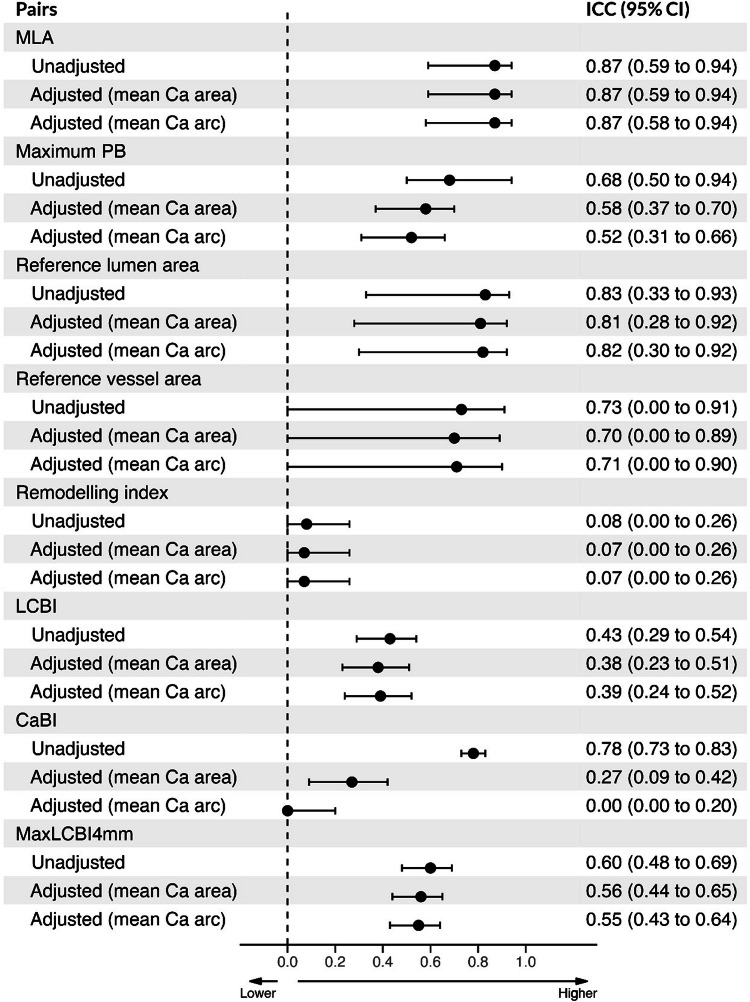
Unadjusted and adjusted (by mean Ca area and mean ca arc) ICC and 95% CI for measurements between NIRS-IVUS and CTA estimated by linear mixed model at lesion level

In total, 304 lesions were detected by NIRS-IVUS and were assessed by CTA and included in the analysis. The estimations of NIRS-IVUS were higher for the MLA, maximum PB and reference lumen and vessel areas; this trend was noted in all the Ca terciles although there was a variation in the median bias and its 95% confident interval for the differences for the above variables in the three groups (Table 3). NIRS-IVUS overestimated the remodelling index in the low and intermediate Ca terciles but there was no difference for the high Ca tercile. The lesion composition analysis results were different in Ca terciles. In the low Ca area tercile, there were differences between CTA and NIRS-IVUS for the CaBI, LCBI, and maxLCBI_4mm_ while, in the intermediate Ca area tercile, CTA underestimated both CaBI and maxLCBI_4mm_ and there was no difference between the two modalities for the LCBI. Finally, in the high Ca area tercile, the CTA estimations were different from NIRS-IVUS for the maxLCBI_4mm_ but similar for the CaBI and LCBI (Table 3). The unadjusted ICC was high between NIRS-IVUS and CTA estimations for the MLA and reference lumen area, and CaBI, moderate for the maximum PB, reference vessel area and maxLCBI_4mm_, and poor for the LCBI and remodelling index. The adjusted mean Ca area or thickness ICC values were not different to the unadjusted for all the studied variables apart from the CaBI whereby the ICC markedly decreased (Fig. 4). The ICC between NIRS-IVUS and CTA estimations in different Ca terciles are presented in Table 2 and Supplementary Fig. 3 while the effect of an increasing mean Ca on the association of NIRS-IVUS and CTA for the lesion level analysis is shown in Fig. 3B. Overall, there was no statistically significant difference between the ICC values reported in different Ca area terciles.

**Table 3:** Lesion-level comparison of the estimations of NIRS-IVUS and CTA in the entire dataset in the low, intermediate, and high Ca area groups

	NIRS-IVUS	CTA	*p*-value *	Median bias (95% CI)
Entire set of studied lesions				
MLA, mm^2^	4.20 (2.46, 6.35)	3.17 (1.96, 5.07)	< 0.001	1.23 (− 0.66, 3.06)
Maximum PB, %	61.56 (51.75, 74.44)	54.61 (35.29, 74.54)	< 0.001	7.00 (− 14.33, 32.36)
Reference lumen area, mm^2^	9.84 (7.44, 12.85)	7.27 (5.44, 10.09)	< 0.001	2.65 (0.34, 5.57)
Reference vessel area, mm^2^	13.80 (10.44, 17.98)	8.73 (6.72, 12.03)	< 0.001	5.16 (1.60, 9.83)
Remodelling index	0.92 (0.78, 1.08)	0.85 (0.59, 1.05)	0.005	0.08 (− 0.58, 0.56)
LCBI	29.50 (0.00, 110.00)	26.66 (0.00, 110.42)	0.279	0.00 (− 168.49, 113.33)
CaBI	104.00 (13.75, 203.25)	60.59 (0.00, 180.78)	< 0.001	15.11 (− 87.87, 125.00)
MaxLCBI_4mm_	173.10 (0.00, 343.81)	88.12 (0.00, 375.00)	0.121	− 0.25 (− 427.50, 290.00)
Low Ca area tercile				
MLA, mm^2^	4.42 (3.09, 6.25)	3.32 (2.37, 5.49)	< 0.001	0.77 (− 0.69, 2.54)
Maximum PB, %	54.22 (49.15, 66.87)	28.13 (19.56, 49.66)	< 0.001	23.46 (1.10, 41.97)
Reference lumen area, mm^2^	8.15 (6.07, 10.36)	6.48 (4.62, 8.14)	< 0.001	2.08 (− 0.06, 4.61)
Reference vessel area, mm^2^	11.25 (8.49, 14.58)	7.52 (5.43, 9.57)	< 0.001	4.52 (1.28, 8.54)
Remodelling index	0.96 (0.84, 1.10)	0.83 (0.65, 1.02)	0.002	0.12 (− 0.47, 0.51)
LCBI	0.00 (0.00, 45.88)	6.67 (0.00, 71.30)	0.045	0 (− 135.00, 54.00)
CaBI	0.00 (0.00, 46.25)	0.00 (0.00, 2.07)	< 0.001	0 (− 3.48, 83.00)
MaxLCBI_4mm_	22.50 (0.00, 181.94)	25.00 (0.00, 187.19)	< 0.001	− 10 (− 437.75, 115.00)
Intermediate Ca area tercile				
MLA, mm^2^	3.71 (2.29, 6.29)	2.98 (1.91, 4.97)	< 0.001	1.11 (− 0.70, 2.94)
Maximum PB, %	62.77 (53.95, 76.91)	53.95 (41.13, 74.21)	< 0.001	6.81 (− 9.40, 30.00)
Reference lumen area, mm^2^	9.84 (7.68, 13.30)	6.97 (5.51, 10.40)	< 0.001	2.76 (0.27, 5.63)
Reference vessel area, mm^2^	13.67 (10.77, 18.17)	8.85 (7.03, 12.21)	< 0.001	5.06 (1.60, 9.10)
Remodelling index	0.90 (0.74, 1.06)	0.76 (0.51, 0.99)	0.015	0.08 (− 0.45, 0.66)
LCBI	39.00 (0.00, 112.00)	13.13 (0.00, 65.36)	0.263	0 (− 153.71, 107. 33)
CaBI	112.00 (60.00, 169.00)	64.34 (35.00, 105.81)	< 0.001	48.51 (− 60.00, 135.00)
MaxLCBI_4mm_	224.00 (0.00, 340.31)	52.50 (10.00, 248.75)	< 0.001	0 (− 323.50, 322.50)
High Ca area tercile				
MLA, mm^2^	4.21 (2.69, 6.41)	3.14 (1.34, 4.66)	< 0.001	1.58 (− 0.18, 3.78)
Maximum PB, %	66.55 (57.60, 75.70)	74.34 (62.65, 83.63)	0.002	− 6.34 (− 20.96, 17.48)
Reference lumen area, mm^2^	11.55 (9.74, 14.86)	8.22 (6.92, 11.00)	< 0.001	3.13 (1.11, 6.60)
Reference vessel area, mm^2^	16.09 (14.02, 21.09)	9.93 (8.31, 13.37)	< 0.001	5.88 (2.78, 11.54)
Remodelling index	0.95 (0.76, 1.08)	0.91 (0.67, 1.25)	0.498	− 0.05 (− 0.74, 0.50)
LCBI	58.00 (8.00, 166.00)	69.47 (17.08, 179.91)	0.275	− 0.613 (− 230.84, 185.00)
CaBI	236.59 (151.00, 340.00)	222.78 (157.43, 319.56)	0.539	26.2 (− 240.43, 171.16)
MaxLCBI_4mm_	223.75 (70.25, 435.00)	220.00 (31.25, 470.00)	< 0.001	− 10 (− 532.50, 326.25)

*CaBI* calcific burden index, *CTA* computed tomography coronary angiography, *LCBI* lipid core burden index, *MaxLCBI*_*4mm*_ maximum LCBI in 4 mm segment, *MLA* minimum lumen area, *NIRS-IVUS* near-infrared spectroscopy intravascular ultrasound, *PB* plaque burden

* *p*-values were obtained by Wilcoxon signed-rank test

The unadjusted proportional odds model showed that NIRS-IVUS had a higher estimated median MLA, maximum PB, reference vessel and lumen area, remodelling index, CaBI and maxLCBI_4mm_ compared to CTA and there was no difference between the two modalities for the LCBI. The adjusted model (by mean Ca area) eliminated the difference between the predictions of the two modalities for the remodelling index and maxLCBI_4mm_ but had no other effect on the other variables. Interaction analysis showed that CTA-derived Ca affected the differences in the estimations of NIRS-IVUS and CTA for the maximum PB, remodelling index, and CaBI (*p*-interaction < 0.001, 0.029 and 0.002, respectively) and there was a trend for the MLA (*p*-interaction = 0.052, Supplementary Fig. 4).

### Cross-sectional-level analysis

In total 23,605 NIRS-IVUS frames were matched with CTA and included in the analysis. The estimations of the two modalities were different for all the studied variables. The results were consistent in all terciles (Table 4).

**Table 4:** Cross-sectional-level comparison of the estimations of NIRS-IVUS and CTA in the entire dataset in the low, intermediate, and high Ca area groups

	NIRS-IVUS	CTA	*p*-value *	Mean bias (95% CI)
Entire set of matched frames				
Lumen area, mm^2^	7.23 (4.90, 10.42)	5.42 (3.66, 8.09)	< 0.001	1.98 (− 2.51, 6.47)
Vessel area, mm^2^	12.88 (8.61, 18.05)	7.74 (5.11, 11.84)	< 0.001	4.95 (− 2.49, 12.39)
Plaque area, mm^2^	5.25 (2.87, 7.89)	1.59 (0.95, 3.32)	< 0.001	2.97 (− 3.03, 8.97)
PB, %	38.65 (27.30, 50.78)	19.08 (16.68, 34.06)	< 0.001	12.39 (− 20.73, 45.50)
LCBI	0.00 (0.00, 0.00)	0.00 (0.00, 0.00)	< 0.001	− 0.01 (− 0.41, 0.39)
CaBI	0.00 (0.00, 0.08)	0.00 (0.00, 0.00)	< 0.001	0.01 (− 0.35, 0.37)
Low Ca area tercile				
Lumen area, mm^2^	6.45 (4.53, 9.64)	5.03 (3.49, 7.61)	< 0.001	1.64 (− 2.38, 5.65)
Vessel area, mm^2^	11.04 (7.62, 16.28)	6.54 (4.49, 10.02)	< 0.001	4.65 (− 2.37, 11.67)
Plaque area, mm^2^	4.31 (2.24, 6.71)	1.21 (0.80, 2.04)	< 0.001	3.01 (− 2.62, 8.64)
PB, %	36.16 (25.29, 47.96)	18.27 (16.08, 19.95)	< 0.001	15.79 (− 15.48, 47.06)
LCBI	0.00 (0.00, 0.00)	0.00 (0.00, 0.00)	< 0.001	− 0.01 (− 0.40, 0.38)
CaBI	0.00 (0.00, 0.00)	0.00 (0.00, 0.00)	< 0.001	0.02 (− 0.22, 0.26)
Intermediate Ca area tercile				
Lumen area, mm^2^	8.67 (6.40, 11.37)	6.20 (4.45, 8.65)	< 0.001	2.53 (− 1.93, 6.99)
Vessel area, mm^2^	13.99 (10.55, 18.52)	8.19 (5.90, 11.43)	< 0.001	6.06 (− 1.04, 13.16)
Plaque area, mm^2^	5.29 (3.04, 7.62)	1.58 (1.09, 2.50)	< 0.001	3.53 (− 2.10, 9.17)
PB, %	35.20 (25.87, 45.71)	18.60 (16.48, 24.65)	< 0.001	13.74 (− 13.49, 40.97)
LCBI	0.00 (0.00, 0.00)	0.00 (0.00, 0.00)	< 0.001	0.01 (− 0.28, 0.30)
CaBI	0.00 (0.00, 0.08)	0.00 (0.00, 0.00)	< 0.001	0.01 (− 0.35, 0.37)
High Ca area tercile				
Lumen area, mm^2^	8.04 (5.38, 11.70)	5.87 (3.67, 8.80)	< 0.001	2.52 (− 2.92, 7.95)
Vessel area, mm^2^	16.97 (12.62, 21.62)	11.95 (8.39, 16.25)	< 0.001	4.77 (− 3.79, 13.33)
Plaque area, mm^2^	8.33 (5.89, 10.68)	5.16 (3.50, 7.86)	< 0.001	2.26 (− 4.85, 9.36)
PB, %	50.15 (39.92, 60.42)	48.27 (36.26, 63.19)	< 0.001	0.20 (− 33.24, 33.65)
LCBI	0.00 (0.00, 0.17)	0.00 (0.00, 0.17)	< 0.001	− 0.02 (− 0.52, 0.48)
CaBI	0.25 (0.08, 0.42)	0.33 (0.17, 0.50)	< 0.001	− 0.05 (− 0.63, 0.54)

*CaBI* calcific burden index, *CTA* computed tomography coronary angiography, *LCBI* lipid core burden index, *NIRS-IVUS* near-infrared spectroscopy-intravascular ultrasound, *PB* plaque burden

* *p*-values were obtained by Wilcoxon signed-rank test

**Fig. 5:**
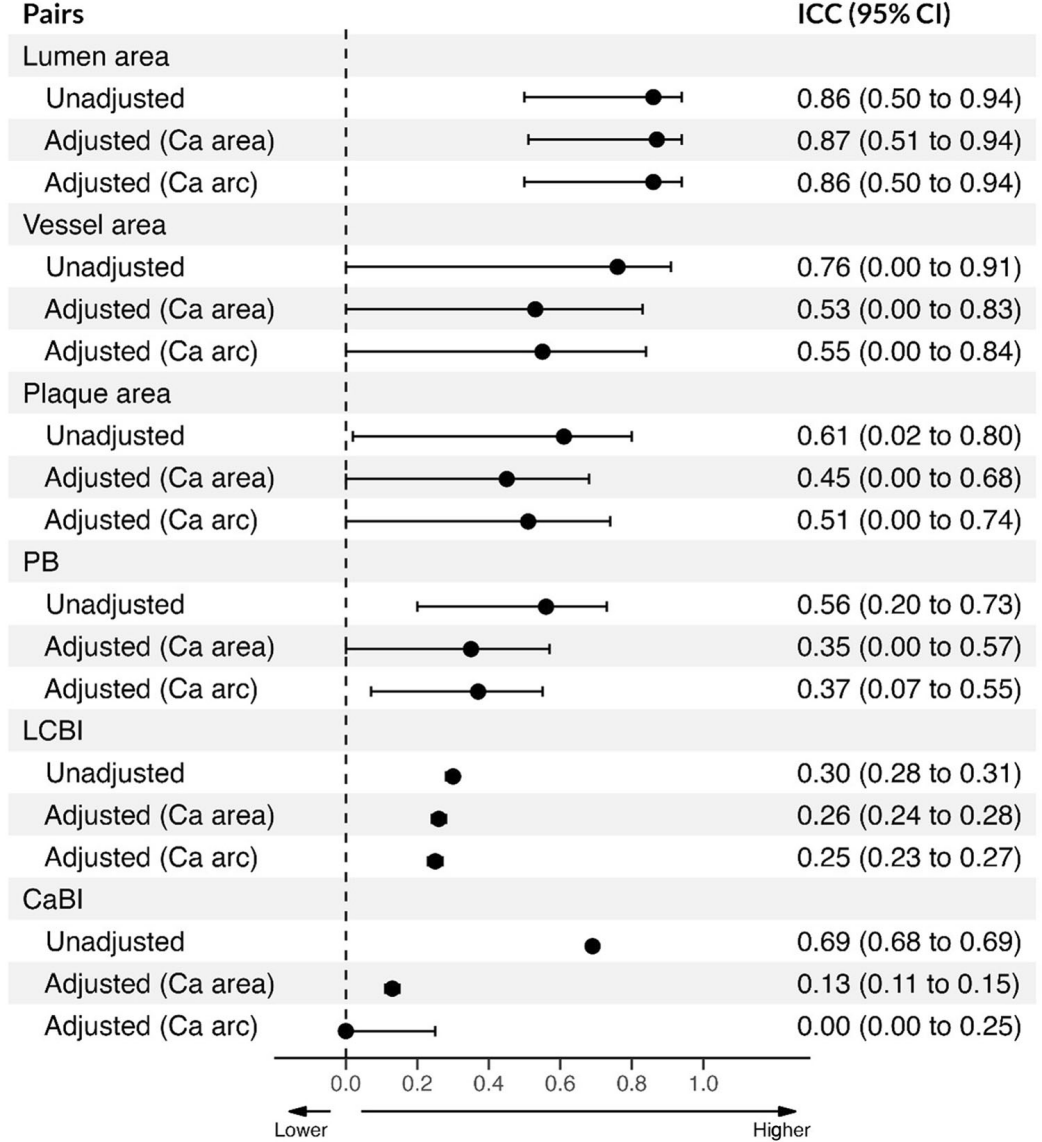
Unadjusted and adjusted (by mean Ca area and mean ca arc) ICC and 95% CI for measurements between NIRS-IVUS and CTA estimated by linear mixed model at the cross-sectional level

Figure 5 shows the ICC between the NIRS-IVUS and CTA estimations. The addition of the Ca area or arc to the ICC had an effect only on the ICC for the LCBI, CaBI suggesting that there was an effect of the Ca burden on the performance of CTA for these metrics. The ICC between the estimations of the two modalities in the three terciles groups are shown in Table 2. A significant heterogeneity was noted between the ICC reported in the three groups for the LCBI, and CaBI indicating an effect of the Ca burden in the agreement of the two modalities for these metrics. CTA estimations for the lipid component seem to correlate better with the NIRS-IVUS in the intermediate Ca tercile while for the CaBI component in the high Ca area tercile (Supplementary Fig. 5). The effect of an increasing mean Ca on the association between NIRS-IVUS and CTA estimations for the cross-sectional level analysis is shown in Fig. 3C.

In both, the unadjusted and adjusted ordinary least square model, NIRS-IVUS was associated with higher estimated MLA, vessel area, plaque area PB and CaBI and lower estimated LCBI. Interaction analysis showed that the CTA-derived Ca area affected the differences in all the estimations of NIRS-IVUS and CTA for the lumen vessel and plaque dimensions and composition (Supplementary Fig. 6).

## Discussion

This is the first study that examined the implications of coronary calcification quantified by CTA on the performance of the modality to assess lumen and plaque and its composition using NIRS-IVUS as the reference standard. We found that the Ca burden (1) did not affect the association of the two modalities for the lumen and plaque dimensions and its composition at segment-level analysis, (2) had no effect on the performance of CTA to quantify the MLA, maximum PB, and characterise its composition, and (3) but it influenced the correlation between CTA and NIRS-IVUS for the lipid and Ca estimations at cross-sectional level analysis. The prediction models built from the matched NIRS-IVUS and CTA data showed that Ca burden (1) had no effect on the agreement of the predicted estimations of NIRS-IVUS and CTA for the lumen and vessel dimensions and composition at the segment level but it affected the PAV estimations (2) influenced the agreement of the predictions of the two modalities at lesion-level for the maximum PB, remodelling index, and CaBI and there was a trend for the MLA, and (3) had a significant effect on the differences between the predictions of NIRS-IVUS and CTA for the lumen vessel and plaque dimensions and compositions.

Ca tissue generates blooming artifacts in CTA which affects data analysis and quantification of lesion severity [16, 17]. Studies examining the implications of coronary calcification on the performance of CTA in evaluating lesion hemodynamic severity using invasive fractional flow reserve (FFR) as a reference standard have shown a negligible effect of Ca burden on the computation of CTFFR [4, 5]. Conversely, there is inconsistency in the findings of studies that examined the effect of Ca on the efficacy of CTA to quantify lumen dimensions and PB against intravascular imaging; this can be at least partially attributed to the limitations of these analyses and more specifically to the small number of patients recruited, the use of early generation CT scanner, their retrospective design and the suboptimal CTA and IVUS co-registration that can cause bias and affect the reported results [1, 18–22].

Our study overcomes these limitations and provides unique insights into the effect of Ca burden on the performance of CTA in assessing lumen and vessel dimensions and quantifying PB and composition. In contrast to other studies, CTA imaging was performed using a 3rd generation CT scanner with a minimum tube voltage of 100 kV—to avoid bias in plaque quantification and effort was made to collect images of optimal quality and reconstruct the CTA using the ADMIRE five iterative reconstruction algorithm—which is not commonly used in clinical practice but appears to be superior to other approaches in the quantification of the PB [7]. Moreover, NIRS-IVUS imaging was performed in the entire coronary tree including the advanced disease and disease-free segments so as to have a representative evaluation of plaque distribution, and after administration of nitrates that were also given prior to CTA imaging, as indicated by the JCCT guidelines [23]. Additionally, we developed a module for accurate CTA and NIRS-IVUS co-registration and matched only the end-diastolic NIRS-IVUS frames—detected retrospectively by a dedicated method—with the CTA so as to avoid errors in the matching that occurred during the back-forward motion of the IVUS probe in the vessel during the cardiac cycle that can affect data co-registration and TAV quantification [13, 24]. Finally, we modified the output of the QAngioCT software and generated spread-out plots of plaque composition to perform a meaningful comparison of the estimations of the two approaches for tissue types.

We found that Ca burden—expressed by the mean plaque area or arc—had no effect on the agreement of the two approaches for the lumen, vessel, TAV, PAV and plaque components at the segment level. Similarly, for lesion-level analysis; Ca burden did not affect the difference between NIRS-IVUS and CTA for the MLA, maximum PB, reference lumen and vessel area, remodelling index and plaque composition. Conversely, the cross-sectional level analysis suggested that the Ca component influenced the ICC between NIRS-IVUS and CTA for the CaBI and LCBI. For the CaBI, the ICC was higher in the high Ca tercile—something that can be explained by the larger range of CaBI values in this group—while for the LCBI the highest value was reported in the moderate Ca tercile. An explanation of the latter finding is the fact that often NIRS detects lipids in disease-free segments with no large NC. Pathological intimal thickening plaque phenotypes can light up in NIRS as they can include lipid tissue and are associated with disease progression, but these lesions are unlikely to have attenuated plaques on CTA [25, 26]. The matched frames included in the intermediate Ca tercile have the atherosclerotic disease and are more likely to portray attenuated plaques in CTA—that are associated with the presence of lipids [27]—while in the high Ca tercile, the blooming artefacts noted in frames with large Ca deposits were likely to mask the surrounding tissues and affect the efficacy of CTA to portray lipid-rich plaques.

Results were different when proportional odds modelling was performed that took advantage of the NIRS-IVUS and CTA estimations to predict the agreement between the two modalities in different Ca patterns. This approach overcomes the limitation of the small sample size and allows a more comprehensive evaluation of the effect of Ca burden on the differences between NIRS-IVUS and CTA estimations. Segment-level analysis demonstrated an effect of the Ca burden on the difference between NIRS-IVUS and CTA for PAV with low Ca area in CTA was related to PAV underestimation that diminishes with the increase of Ca (Supplementary Fig. 3). The lesion-level analysis also showed a significant effect of Ca area on the difference between NIRS-IVUS and CTA for the PB (CTA underestimated PB in lesions with low calcification and overestimated PB in these with increased Ca burden), remodelling index (CTA underestimated remodelling index in lesions with low Ca but provided similar estimation to NIRS-IVUS in Ca lesions) and CaBI (CTA overestimated Ca burden in lesions with increased Ca) and there was a trend for the MLA with CTA tending to underestimate the MLA in lesions with increased calcification. Finally, the cross-sectional level analysis revealed a significant interaction between the Ca area and the NIRS-IVUS and CTA estimations; a large Ca area was associated with a larger underestimation of the lumen area and overestimation of the vessel and plaque area, PB, CaBI and LCBI. These findings should be attributed to the limited efficacy of CTA to assessing soft fibrotic-rich plaques as shown in histology studies and the blooming artefacts that are seen in large Ca deposits that mask the lumen, causing an overestimation of PB and CaBI which impedes the accurate quantification of the low attenuation component [28].

From the above, it is apparent that a large Ca component may impose challenges in the accurate quantification of plaque pathology. Machine learning methodologies trained from intravascular imaging data may be useful in overcoming the limitations of current CT scanners with the first studies in the field showing promising results—however, prospective validation in large datasets is needed before advocating their use in routine practice [11]. In parallel, advances in CT scanners are expected to improve CTA image quality reduce the blooming effects of Ca and enhance its potential in clinical practice and research [29, 30]. Indeed, cumulative data indicate that photon counting CT enables a more accurate assessment of the lumen dimensions and quantification of the Ca burden in calcified lesions and outperforms conventional CTA in the evaluation of stented segments where the increased blooming artefacts limit lumen visibility [31–33].

### Limitations

Despite the fact that this study is the largest in the field assessing in a comprehensive fashion the effect of Ca deposits on the performance of current CTA technologies to evaluate plaque pathology, it has limitations that should be acknowledged. Firstly, it is a single centre study conducted using a single CT scanner; it is, therefore, unclear whether the findings of this analysis apply to CTA data collected by other CT systems and reconstructed using different reconstruction approaches (that also appear to affect the quantification of the Ca volume) [34]. Secondly, despite that this is one of the largest studies in the field the number of the included patients and studied segments may have not provided sufficient power to thoroughly assess the effect of Ca on the agreement between the estimations of NIRS-IVUS and CTA. The use of proportional odd modelling may allow us to partially overcome this restraint, but it also has limitations as it is associated with a larger systematic bias than the assessment of the two methods’ agreement. Thirdly, the severity of lesion calcification was based on the estimations of CTA. It is well known that CTA is unable to detect small Ca deposits, and this is likely to have affected the reported results; however, it was preferred over NIRS-IVUS estimation as IVUS does not allow quantification of Ca volume because of the acoustic shadowing behind Ca tissue [35]. The latter did not allow volumetric comparison of intravascular and CTA and NIRS-IVUS estimations of the Ca tissue as it has been performed in recently reported OCT studies [2, 3]. Nevertheless, the use of NIRS-IVUS in this study has allowed us to evaluate, for the first time the implications of Ca burden on the CTA on the computation of lipid component. Finally, there was a significant difference in the length of the segment of interest between NIRS-IVUS and CTA (Δ_length_: − 3.49 ± 4.62 mm, *p* < 0.001); this may have affected the agreement between NIRS-IVUS and CTA for the volumetric analyses (i.e. lumen, vessel and TAV) at segment-level but not the lesion- and frame-level analyses. Moreover, this difference is unlikely to have affected the impact of the Ca burden on the agreement between NIRS-IVUS and CTA for the above variables as the difference in the length of the segment of interest was consistent in the three Ca terciles and it was not associated with the Ca burden (*p* = 0.215).

## Conclusions

The efficacy of CTA imaging to assess the extent, severity, and compositional characteristics of coronary atheroma can be affected by Ca burden on CTA images. This should be taken into account in studies using CTA to assess plaque vulnerability and in revascularisation planning.

## Supplementary information

ELECTRONIC SUPPLEMENTARY MATERIAL

## Abbreviations

BA: Bland–Altman
Ca: Calcific
CaBI: Calcific burden index
CAD: Coronary artery disease
CTA: Computed tomography coronary angiography
FF: Fibrofatty
FFR: Fractional flow reserve
FT: Fibrotic tissue
ICC: Intra-class correlation coefficients
LCBI: Lipid core burden index
LOA: Limits of agreements
MaxLCBI_4mm_: Maximum LCBI in 4 mm segment
MLA: Mean lumen area
NC: Necrotic core
NIRS-IVUS: Near-infrared spectroscopy-intravascular imaging
PAV: Percentage atheroma volume
PB: Plaque burden
PCI: Percutaneous coronary intervention
TAV: Total atheroma volume
