# Supplementary material for: Implications of coronary calcification on the assessment of plaque pathology: a comparison of computed tomography and multimodality intravascular imaging
Source: Eur Radiol. 2024 Aug 22;35(4):1745–60. doi: 10.1007/s00330-024-10996-x (PMC11914240; doi:10.1007/s00330-024-10996-x)
Supplement: Supplementary file 1 — ELECTRONIC SUPPLEMENTARY MATERIAL [file 330_2024_10996_MOESM1_ESM.pdf]

Implications of coronary calcification on the assessment of plaque pathology: a comparison of computed tomography and multimodality intravascular imaging

ELECTRONIC SUPPLEMENTARY MATERIAL

Eur Radiol (2024) Yap NAL, Ramasamy A, Tanboga IH, et al.

Supplementary Methods

CTA and NIRS-IVUS imaging

Computed tomography coronary angiography (CTA) imaging was undertaken using a dual-source CT scanner (Somatom Force, Siemens Healthineers). All patients received sublingual nitroglycerin (400micrograms) while those with increased heart rate of >70 beats per minute received metoprolol (maximum 40mg) provided there were no contraindications. CTA imaging was performed using a prospective ECG-triggered sequential scan mode, a gantry rotation time of 250ms, 128 x 2 x 0.5mm collimation with z-flying focal spot for both detectors and a minimum tube voltage of 100kV defined by the CarekV algorithm whereas the tube current was derived by the scanner.

Near-infrared spectroscopy-intravascular (NIRS-IVUS) imaging was performed in all the 3 major epicardial vessels and their side branches with a diameter ≥2mm after intracoronary injection of 400micrograms of nitrates. A 2.4F MakotoTM NIRS-IVUS 35-65MHz Imaging System (Infraredx) was advanced to the distal end of the artery and then pulled-back at a speed of 0.5mm/s using an automated pull-back device. Lesion pre-dilatation was performed with a 2mm balloon, before NIRS-IVUS imaging only in cases that the probe could not cross the lesion.

CTA data reconstruction

The CTA data were reconstructed applying a medium smooth kernel (b40f), a slice thickness 0.50mm with 0.30mm increments and a highest strength model-based iterative reconstruction (ADMIRE 5) algorithm – this approach was selected, as a recent study has shown that it is superior to other reconstruction approaches in quantifying the extent and severity of coronary artery disease (CAD).[[1]] The reconstructed images were reviewed by two expert analysts who assessed image quality using the Likert score; data with excellent, good, or moderate quality were included in the analysis, while those with poor were excluded.[[2]]

Co-registration of CTA and NIRS-IVUS imaging data

A dedicated software was used to co-register the CTA and NIRS-IVUS data corresponding to the segment of interest (QAngioCT IVUS Matcher, Medis Medical Imaging) [[1]]. This allows

simultaneous display of CTA and NIRS-IVUS images and enables identification of anatomical landmarks – such as the coronary ostia or the origin of side branches – that are seen in both modalities to match CTA and NIRS-IVUS. Linear interpolation was then applied to co-localise sections located between these landmarks. In this way each segmented CTA frame was matched to an analysed NIRS-IVUS cross-section.

Supplementary Table 1. Clinical characteristics of the studied patients.

	**Studied patients (n=64)**
Age (years)	61.7 (53, 70)
Gender (male)	51 (79.7%)
Current smoker	4 (6.3%)
Family history of CAD	40 (62.5%)
**Co-morbidities**	
Diabetes mellitus	22 (34.4%)
Hypertension	35 (54.7%)
Hypercholesterolemia	45 (70.3%)
Renal failure*	4 (6.3%)
Previous PCI	14 (21.9%)
LV function	
Good LV function	60 (93.8%)
Impaired LV function**	4 (6.2%)
**Studied vessels by NIRS-IVUS**	
Total number of vessels	186
LAD/diagonal branches	64 (34.4%)
LCx/intermediate/obtuse marginal branches	78 (41.9%)
RCA	44 (23.7%)

Table footnote: CAD, coronary artery disease; LAD, left anterior descending artery; LCx, left circumflex artery; LV, left ventricle; NIRS-IVUS, near-infrared spectroscopy–intravascular ultrasound; PCI, percutaneous coronary intervention; RCA, right coronary artery.

*Renal failure is defined as an estimated glomerular filtration rate of <60ml/min/1.73m^2

**Impaired LV function is defined as LV ejection fraction of <50%.

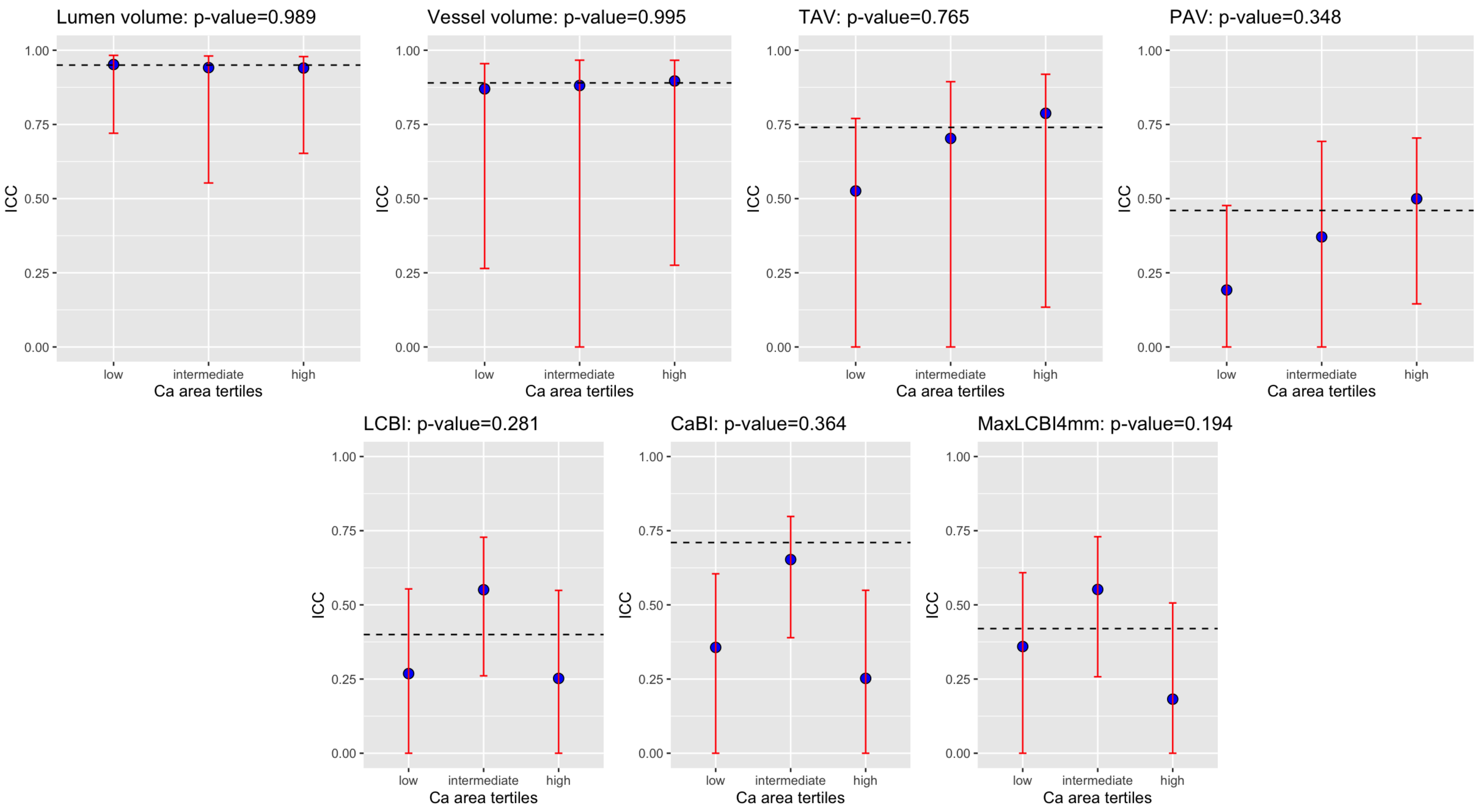

Supplementary Figure 1. ICC and 95% CI for measurements between NIRS-IVUS and CTA according to Ca area tertiles at the segment level. Horizontal black dashed line indicates overall ICC.

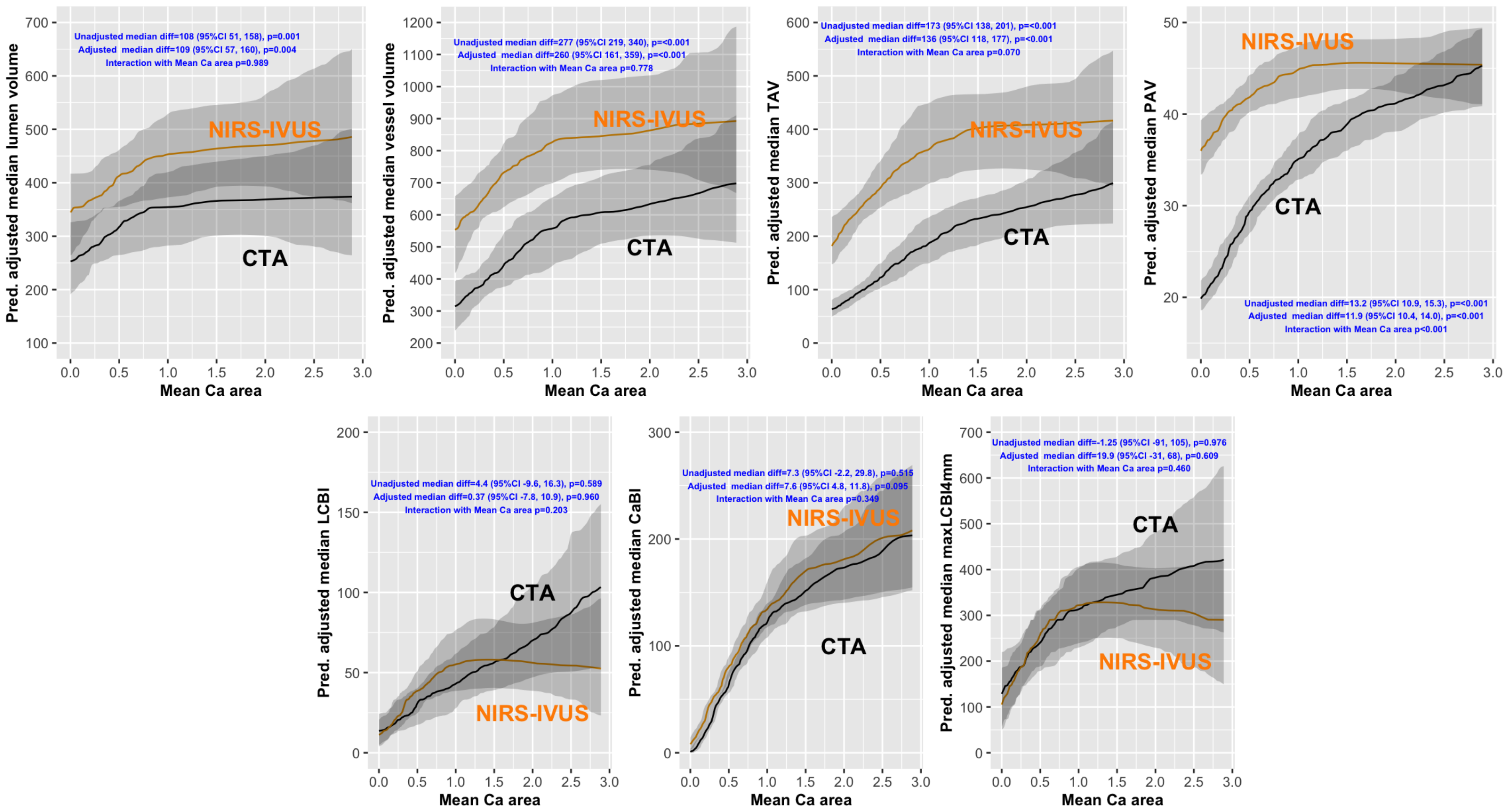

Supplementary Figure 2. The partial effect of Ca on the predicted median measurements by NIRS-IVUS and CTA estimated by proportional odds model at the segment-level. Black and orange solid line indicate CTA and NIRS-IVUS. Unadjusted and adjusted predicted median difference between modalities were noted with p-value.

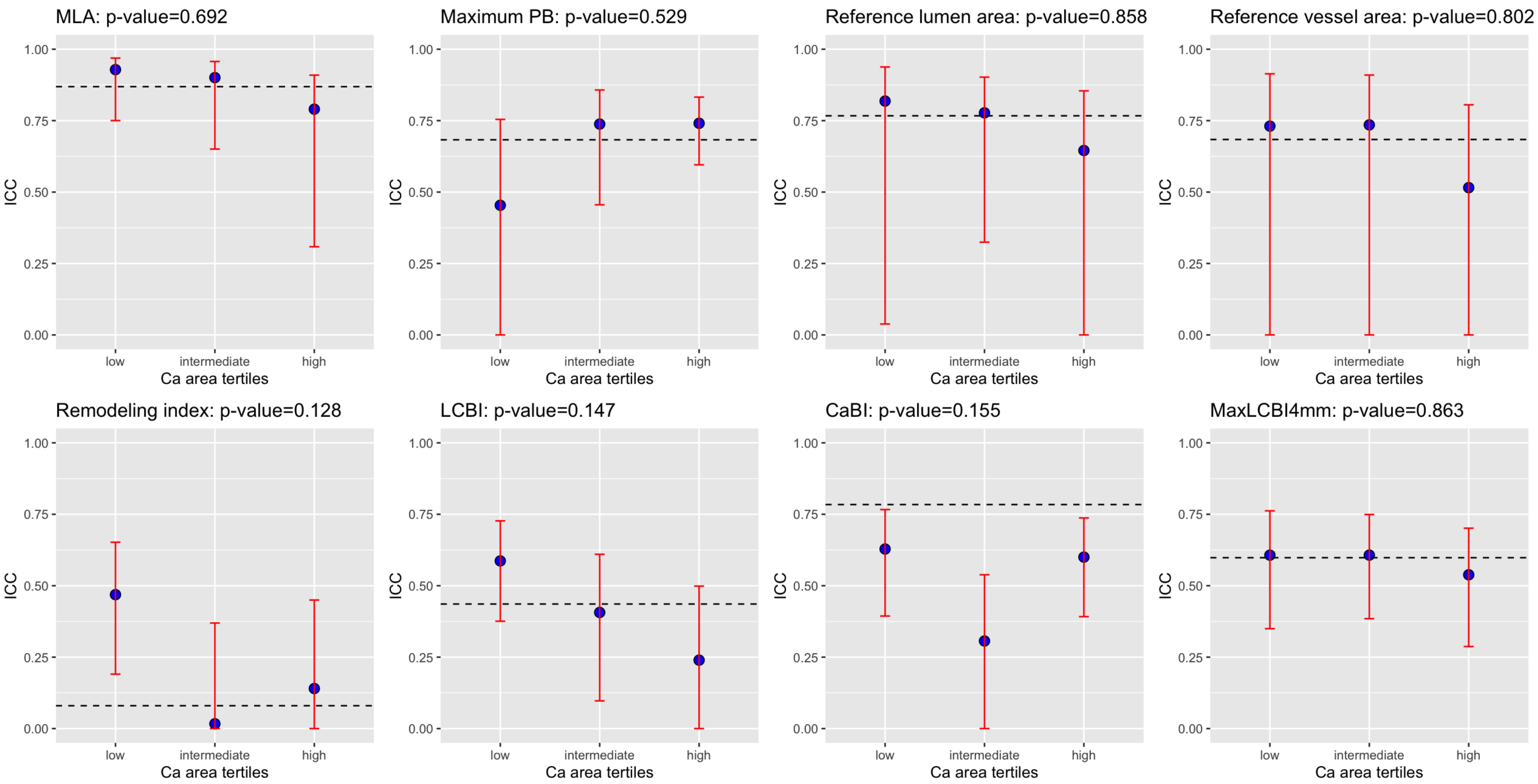

Supplementary Figure 3. ICC and 95% CI for measurements between NIRS-IVUS and CTA according to Ca area tertiles at the lesion level. Horizontal black dashed line indicates overall ICC.

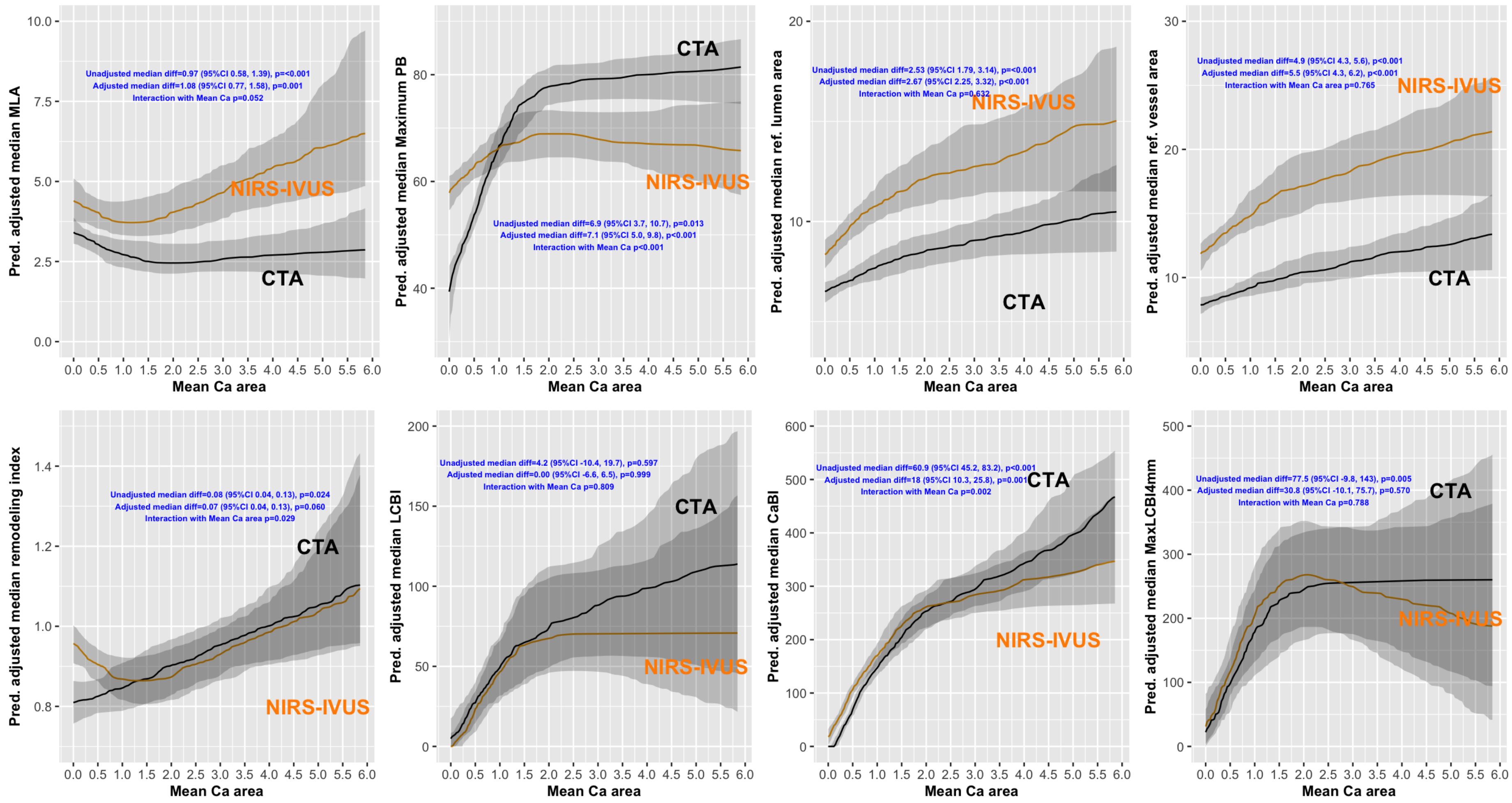

Supplementary Figure 4. The partial effect of Ca on the predicted median measurements by NIRS-IVUS and CTA estimated by proportional odds model at the lesion level. Black and orange solid line indicate CTA and NIRS-IVUS. Unadjusted and adjusted predicted median difference between modalities were noted with p-value.

Eur Radiol (2024) Yap NAL, Ramasamy A, Tanboga IH, et al.

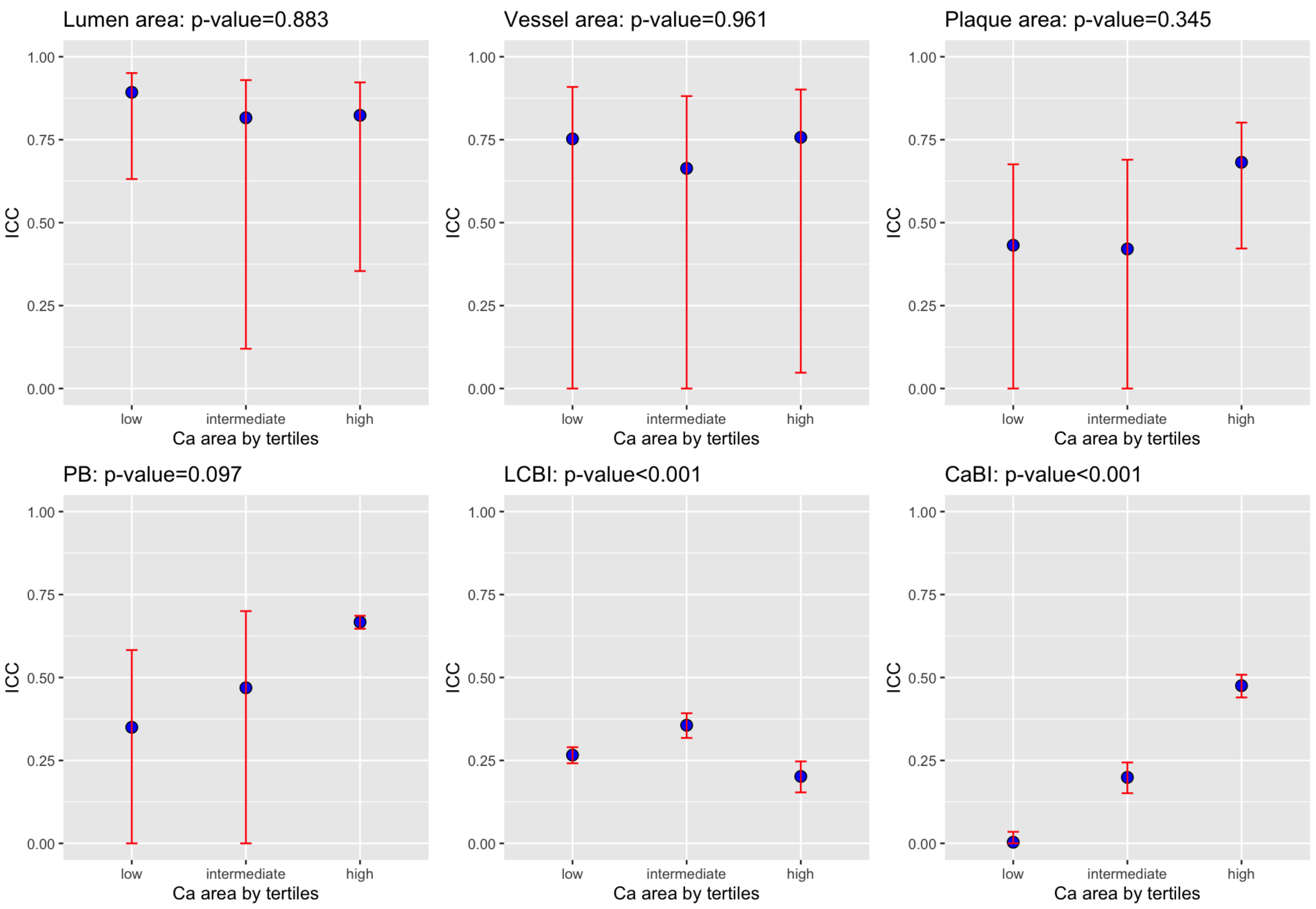

Supplementary Figure 5. ICC and 95% CI for measurements between NIRS-IVUS and CTA according to Ca area tertiles at the frame level. Horizontal black dashed line indicates overall ICC.

Eur Radiol (2024) Yap NAL, Ramasamy A, Tanboga IH, et al.

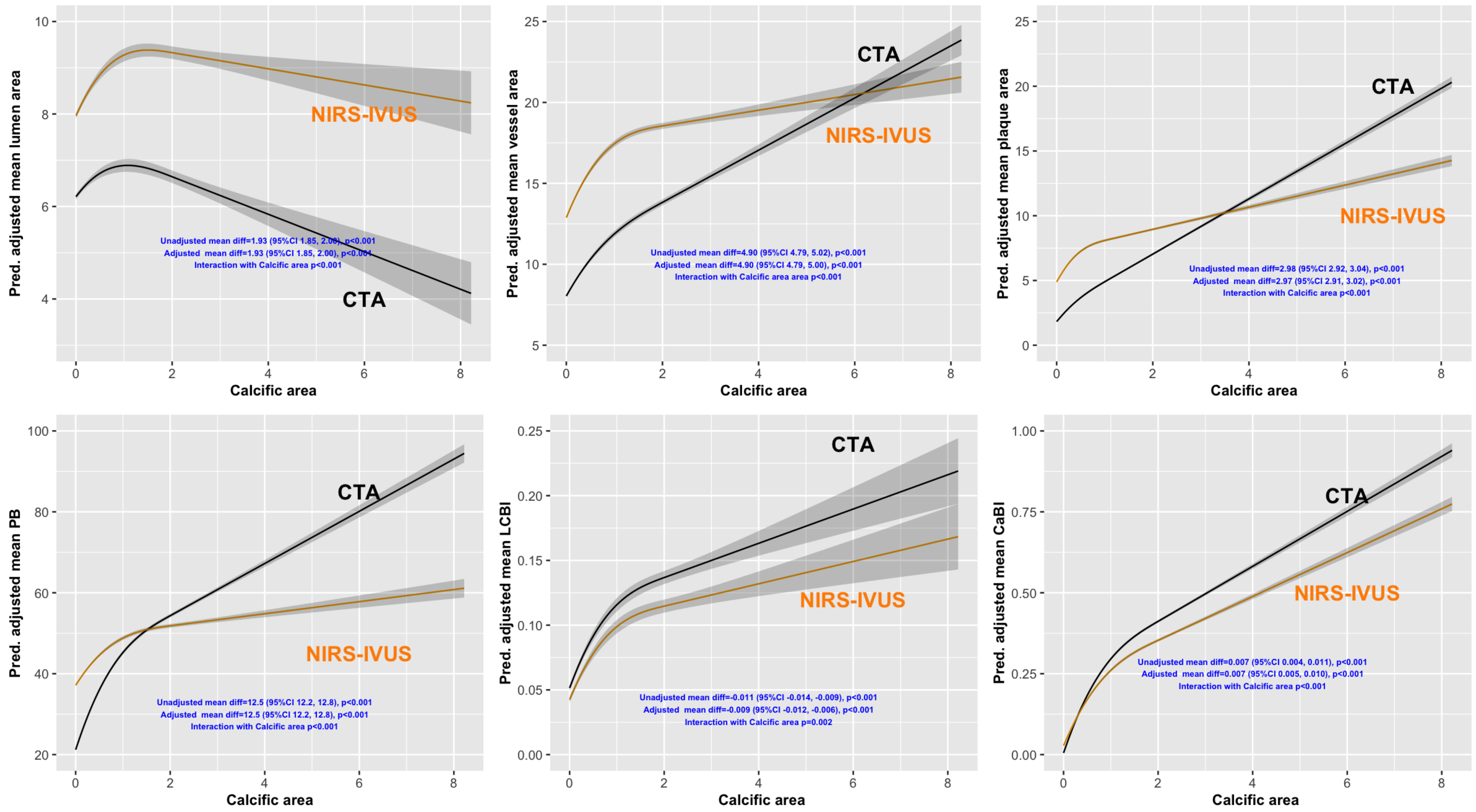

Supplementary Figure 6. The partial effect of Ca on the predicted median measurements by NIRS-IVUS and CTA estimated by ordinary least square model at the frame level. Black and orange solid line indicate CTA and NIRS-IVUS. Unadjusted and adjusted predicted median difference between modalities were noted with p-value.

References

1. Ramasamy A, Hamid AKA, Cooper J, et al. Implications of computed tomography reconstruction algorithms on coronary atheroma quantification: Comparison with intravascular ultrasound (2023) *J Cardiovasc Comput Tomogr*. DOI:10.1016/j.jcct.2022.09.004
2. Pontone G, Bertella E, Mushtaq S, et al. Coronary artery disease: diagnostic accuracy of CT coronary angiography--a comparison of high and standard spatial resolution scanning (2023) *Radiology*. DOI:10.1148/radiol.13130909
